# Global monitoring of the impact of the COVID-19 pandemic through online surveys sampled from the Facebook user base

**DOI:** 10.1073/pnas.2111455118

**Published:** 2021-12-13

**Authors:** Christina M. Astley, Gaurav Tuli, Kimberly A. Mc Cord, Emily L. Cohn, Benjamin Rader, Tanner J. Varrelman, Samantha L. Chiu, Xiaoyi Deng, Kathleen Stewart, Tamer H. Farag, Kristina M. Barkume, Sarah LaRocca, Katherine A. Morris, Frauke Kreuter, John S. Brownstein

**Affiliations:** ^a^Division of Endocrinology, Boston Children’s Hospital, Boston, MA 02115;; ^b^Computational Epidemiology Lab, Boston Children’s Hospital, Boston, MA 02115;; ^c^Harvard Medical School, Boston, MA 02115;; ^d^Broad Institute of Harvard and MIT, Cambridge, MA 02142;; ^e^Department of Epidemiology, Boston University, Boston, MA 02118;; ^f^Joint Program in Survey Methodology, University of Maryland, College Park, MD 20742;; ^g^Center for Geospatial Information Science, University of Maryland, College Park, MD 20742;; ^h^Meta, Menlo Park, CA 94025;; ^i^Department of Statistics, Ludwig-Maximilians-Universität, Munich 80539, Germany

**Keywords:** COVID-19 surveillance, global health, human social sensing, SARS-CoV-2 testing

## Abstract

Simultaneously tracking the global impact of COVID-19 is challenging because of regional variation in resources and reporting. Leveraging self-reported survey outcomes via an existing international social media network has the potential to provide standardized data streams to support monitoring and decision-making worldwide, in real time, and with limited local resources. The University of Maryland Global COVID-19 Trends and Impact Survey (UMD-CTIS), in partnership with Facebook, has invited daily cross-sectional samples from the social media platform's active users to participate in the survey since its launch on April 23, 2020. We analyzed UMD-CTIS survey data through December 20, 2020, from 31,142,582 responses representing 114 countries/territories weighted for nonresponse and adjusted to basic demographics. We show consistent respondent demographics over time for many countries/territories. Machine Learning models trained on national and pooled global data verified known symptom indicators. COVID-like illness (CLI) signals were correlated with government benchmark data. Importantly, the best benchmarked UMD-CTIS signal uses a single survey item whereby respondents report on CLI in their local community. In regions with strained health infrastructure but active social media users, we show it is possible to define COVID-19 impact trajectories using a remote platform independent of local government resources. This syndromic surveillance public health tool is the largest global health survey to date and, with brief participant engagement, can provide meaningful, timely insights into the global COVID-19 pandemic at a local scale.

## Significance

The University of Maryland Global COVID Trends and Impact Survey (UMD-CTIS), launched April 2020, is the largest remote global health monitoring system. This study includes ∼30 million responses through December 2020 from all 114 countries/territories with survey weights to adjust for nonresponse and demographics. Using self-reported cross-sectional survey data sampled daily from Facebook users, we confirm consistent demographics and COVID-19 symptoms. Our global model predicts local COVID-19 case trends. Importantly, one survey item strongly correlates with reported cases, demonstrating potential utility in locales with scant UMD-CTIS sampling or government data. Despite limitations resulting from sampling, nonresponse, coverage, and measurement error, UMD-CTIS has the potential to support existing monitoring systems for COVID-19 as well as other new as-yet-undefined global health threats.

In December 2019, the COVID-19 pandemic swept across the globe and challenged the scientific community to urgently assess and intervene (1). While impressive public health mitigation efforts have been made, such as the expansion of case testing and reporting infrastructure, nonpharmaceutical interventions, and rapid vaccine development, barriers remain to understanding and controlling the pandemic (2). The lack of preexisting knowledge of the SARS-CoV-2 virus and lack of uniform COVID-19 data among and within countries has reduced our ability to assess severity and direct action across regions (3). This data issue may have been additionally challenging in areas with less resilient healthcare systems, where disease surveillance may be delayed or underestimated and surge capacity limited (4, 5). This lack of timely information may obscure the actual impact during a crucial window to mobilize targeted support (6).

One successful approach to responding to the need for timely trends and insights has been to leverage syndromic surveillance through surveys (7–12). Previous work on influenza, and now COVID-19, has demonstrated the utility of this approach based on self-reported symptoms and other data, including burden estimation, hot-spot identification, and mitigation tracking (13–16), in near real-time. While platforms differ in their data collection methodology, scope, and target population, insights have been comparable and used to inform decision-making (17–19). This further underscores the value of participatory epidemiology in the pandemic setting.

While existing platforms have many strengths, such as longitudinal disease trajectories (9, 20) and rapid deployment of timely survey questions (11, 21), there is a need for sufficient data from diverse populations for certain research questions, especially from regions already struggling with the pandemic response. High-quality, prospective, opt-in platforms that rely on government endorsement, advertising, or word of mouth may not be feasible given low uptake and high attrition in conjunction with the incidence of disease. They may also be particularly problematic in populations with limited government trust or variable access to smartphone technology.

Using a recruitment mechanism from a massive social media platform sampling frame—the Facebook Active User Base (FAUB)—the University of Maryland (UMD) developed the Global COVID-19 Trends and Impact Survey (UMD-CTIS) in collaboration with Facebook (22). The UMD-CTIS data stream combines user responses to a unified survey instrument with survey weights adjusting for sampling, nonresponse, and basic demography for 114 countries and territories worldwide. Because the FAUB spans locales with varied languages, social structures, and economic resources, UMD-CTIS could provide, with its daily samples, a unique, fine-grained understanding of the longitudinal development of global and region-specific health at a scale not feasible previously (23). The ability to pool global UMD-CTIS data could enable a timely glimpse of emerging syndromes. Additionally, the innovative UMD-CTIS approach of leveraging sampled users as community sensors (24) could enhance local surveillance signals with fewer responses.

We sought to characterize the utility of UMD-CTIS as a global and local public health monitoring resource for these fundamental syndromic surveillance applications. Toward this end, we used 31 million UMD-CTIS responses from before COVID-19 vaccine deployment to evaluate if the unique study design produces consistent, cross-sectional views over time, first of local demographics and then of the early COVID-19 waves. We test if combined data from all countries/territories can be used to identify known symptom predictors of testing positive for COVID-19. Finally, we describe how UMD-CTIS signals, using symptoms and human social sensing, may improve syndromic surveillance for COVID-19.

## Results

### Study Participants.

**Fig. 1.**
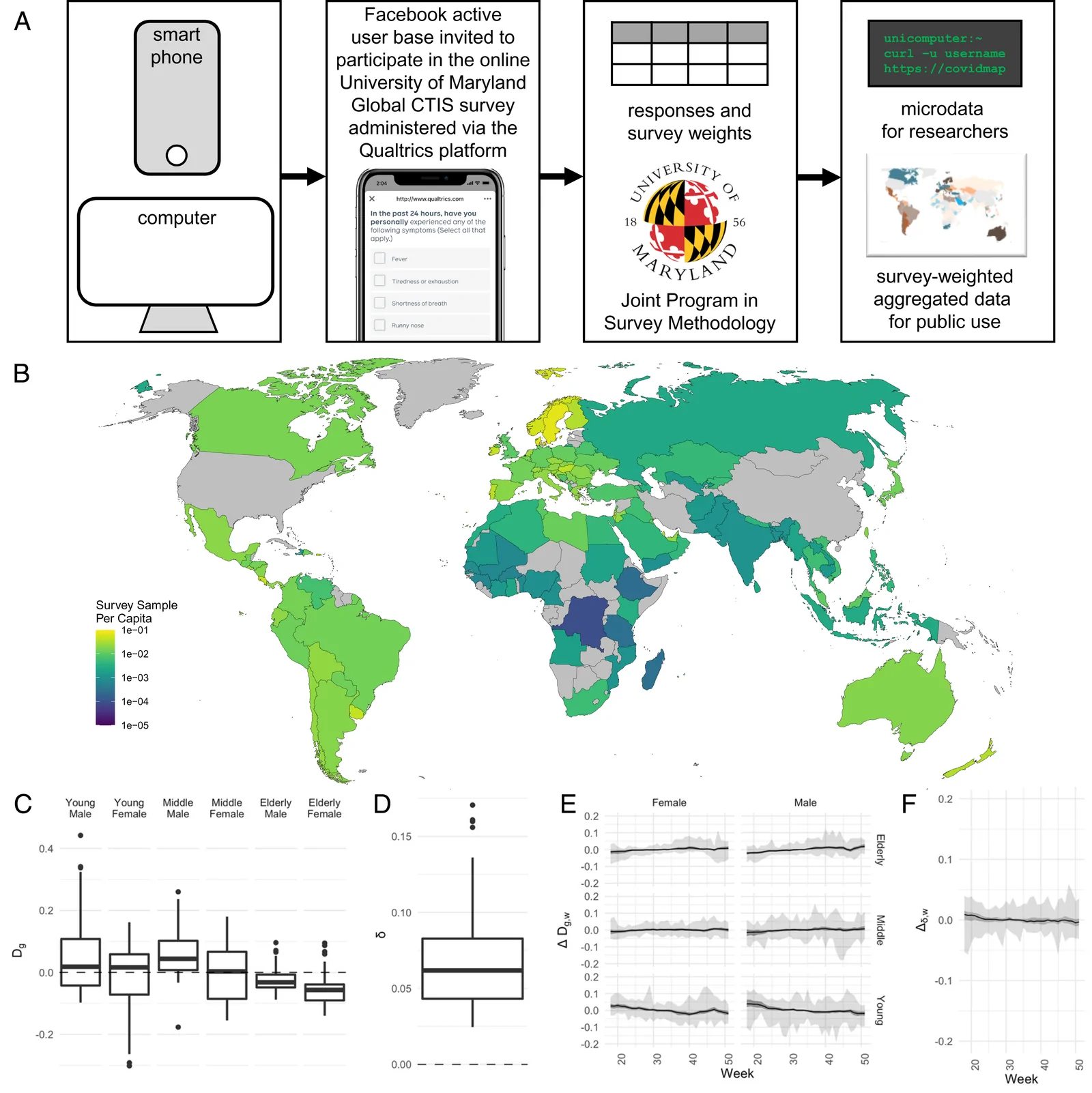
UMD-CTIS data pipeline, coverage, and demographic distributions. (*A*) The FAUB is sampled daily and invited to participate in the online UMD-CTIS, administered by Qualtrics and accessed via an online form using a smartphone or computer. Participants are asked about demographics, COVID-19 symptoms, behaviors, and outcomes. Facebook supplies survey weights to account for nonresponse and to adjust for basic demographics of the participant. Aggregated data are released to the public in near real time. Researchers may apply to use raw microdata to study COVID-19. (*B*) The map of the distribution of surveys per capita during the study period for countries and territories sampled and that have survey weights (*n* = 114, gray for all other countries/territories). (*C*) The distribution of difference of proportion in each group in UMD-CTIS versus local demographics by six age–gender groups (male and female versus young [18 to 34 y], middle [35 to 54 y], and elderly [>54 y]), that is, *D_g_* = *P_g,UMD-CTIS_* − *P_g,Census_*, where *P_g_* is the proportion in group *g*. (*D*) The distribution of mean absolute differences across age–gender groups (i.e., δ = *Σ*_*g*_ |*D_g_*|/6). The distribution by week (*w*) of the change (*Δ*) in the (*E*) difference of proportions [*ΔD*_*g,w*_ = *D_g,w_* – median(*D_g,w_*)] and (*F*) mean absolute difference [*Δ*_δ_*_,w_* = δ*_w_* – median(δ*_w_*)] versus the median measure for that country/territory over the study period. The range across all locales (light ribbon), 25th to 75th percentile (dark ribbon), and median (solid line) are shown.

The UMD-CTIS (summarized in Fig. 1*A*) includes 127 self-reported time-varying COVID-19–related measures, including key demographic, behavior, and health impacts, at an unparalleled spatiotemporal scale. The survey has been running daily since April 23, 2020. In this analysis, we use data from the first 34 complete weeks of the survey, ending just prior to the COVID-19 vaccine rollout (International Organization for Standardization [ISO] weeks 18 to 51 or April 27 to December 20, 2020). The same questionnaire was fielded in all countries as a web-based instrument. Over the course of the study period reported here, six versions of the survey were deployed to Facebook users sampled from the FAUB (survey questions and changes summarized in *SI Appendix*, Table 1). Questions were translated into 56 languages (25), which excludes only about 5% of the FAUB globally (estimates by region and population not available). There were 31,142,582 responses with survey weights. The responses were from 114 countries and territories, with a median [interquartile range (IQR)] number of responses of 7,837 (6,312, 9,458) surveys per week per locale (survey uptake maps in Fig. 1*B* and *SI Appendix*, Fig. 1). Here, the terms “local” or “locale” may be used to refer to individual countries and territories in UMD-CTIS. Countries and territories without survey weights (*n* = 136) and in which Facebook is not allowed to operate are not included. Data from the United States are also not included because a slightly different questionnaire had been used there at the beginning of the study, and the sampling methodology is different in the US CTIS compared to UMD-CTIS (26). The questionnaires were purposefully kept short to reduce respondent burden and possible drop-off (mean response time 9.2, median [IQR] 7.2 [5.3, 10.1] min).

### Demographic Characteristics of Respondents by Country/Territory and Time.

The UMD-CTIS had several potential sources of bias. For example, as an internet-based social media user group, the FAUB is a nonrandom sample of the population in the countries/territories covered by UMD-CTIS. In addition, the response to invitations to participate in the survey and the response to individual survey items may vary by user. Nevertheless, this very large, globally distributed user base, in conjunction with the UMD-CTIS sampling scheme and repeated cross-sectional surveys, could provide a valuable COVID-19 data stream for public health trends—provided these biases are consistent over relevant timeframes and the signals from respondents adequately capture relevant population-wide epidemiology. We therefore sought to evaluate the demographic composition that UMD-CTIS achieved across locales despite these potential sources of bias. We compared UMD-CTIS with the census population (27) for six age–gender groups (i.e., *D_g_* = *P_g,_*_UMD-CTIS_ − *P_g,_*_Census_, in which *P_g_* is the proportion in group *g*, Fig. 1*C*). Across all locales, the difference was more often positive for male groups and negative for elderly groups (median *D_g_* for young females 0.016, middle females 0.003, elderly females −0.057, young males 0.018, middle males 0.044, and elderly males −0.032). Male respondents are more frequent in Africa and Asia, in contrast to the documented female predominance of other health research studies. This may reflect regional differences in technology access and use (28, 29). To quantify the overall differences, we combined the age–gender group differences to estimate the mean absolute difference (i.e., δ = *Σ*_*g*_ |*D_g_*|/6) for each locale (Fig. 1*D*). The median δ was 0.062, and the majority had a δ < 0.1 (84%, *n* = 94/112). Thus, for many countries and territories, UMD-CTIS demographics prior to application of survey weights were similar to country/territory census demographics.

**Fig. 2.**
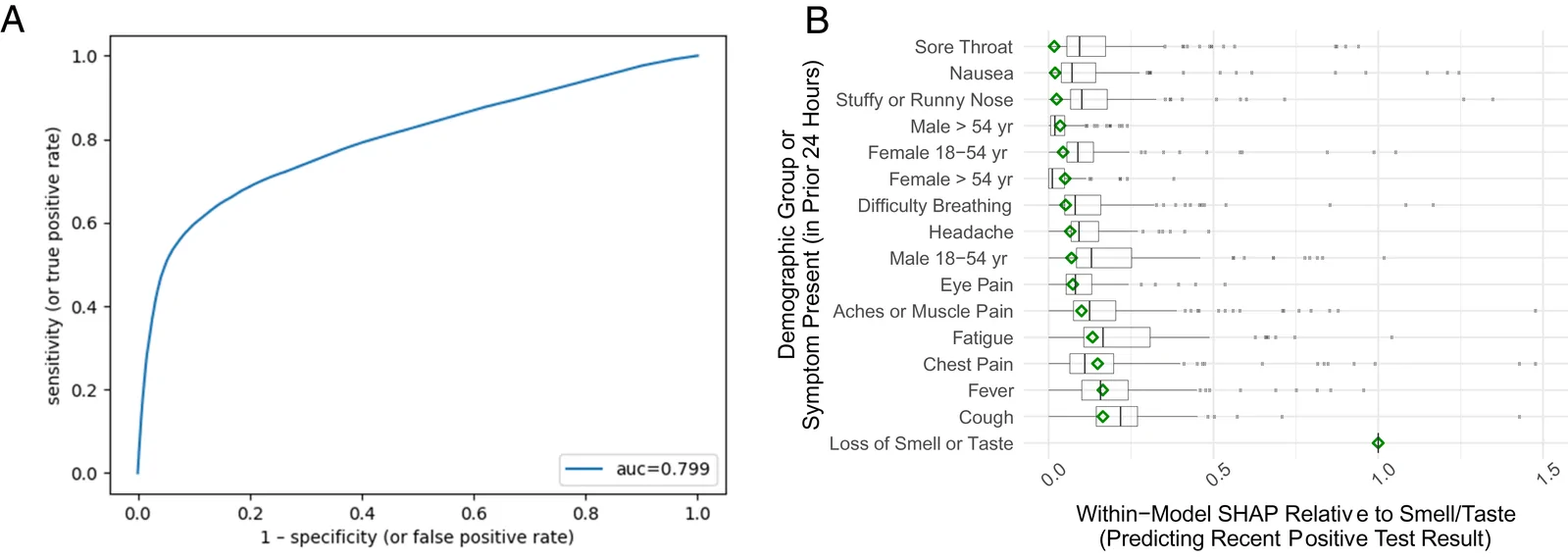
The global model predicting recent COVID-19 positive test results using self-reported symptoms and minimal demographic data. (*A*) The receiver operating characteristic of the hyperparameter tuned global model showing the area under the curve. (*B*) The SHapley Additive exPlanations distribution of relative feature importance from the global model (green diamonds) compared to country/territory models (box and whisker plots). The within-model feature importance was normalized to loss of smell/taste to facilitate between-model comparison.

UMD-CTIS time trends could be particularly valuable for COVID-19 epidemiology, such as community transmission, testing barriers, socioeconomic insecurity, knowledge, practices, and mitigation measures (21, 23, 30, 31). To understand time trends in UMD-CTIS respondents and how this might affect the interpretation of epidemiologic trends, we sought to evaluate weekly variability in UMD-CTIS demographics. For each country/territory and week (*w*), we quantified the demographic variability as the change in the age–gender difference measure versus the median for that country/territory (i.e., *Δ*_*Dg*,w_ = *D_g,w_* – median[*D_g,w_*], Fig. 1*E*). The middle age groups were the least variable over the study period, while the young male group varied more from the median in the first weeks of the study. To quantify the week-to-week variability of overall differences for each locale, we calculated the change in the mean absolute difference versus the median for that country/territory (*Δ*_δ_*_,w_* = *δ_w_* – median[*δ_w_*], Fig. 1*F*). The demographic composition of UMD-CTIS respondents over time was relatively stable (range *Δ*_δ_*_,w_* −0.057 to 0.059 in all country/territory weeks). The extended period covering survey version 5 was broadly the most consistent with respect to this measure. The beginning of the study had the most variability (week 18, median *Δ*_δ_*_,18_* across all locales was 0.009). Certain countries and territories had substantial (e.g., Haiti) or minimal (e.g., Japan) demographic variability over time (*Δ*_δ_*_,w_* by country/territory in *SI Appendix*, Fig. 2). Characterizing the variability of all UMD-CTIS covariates was beyond the scope of this analysis. Nevertheless, since the demographic questions were at the end of the survey during this study period, the variability in demographics over time provides one metric for understanding time trends and demographic consistency among UMD-CTIS respondents. Despite the potential bias in the FAUB sampling frame, sampling scheme, survey nonresponse, and/or response to demographic questions, the survey design resulted in demographic consistency for most locales for most of the study period.

### Using Global Surveillance Data to Derive Symptoms Predictive of Individual COVID-19–Positive Test Result.

**Fig. 3.**
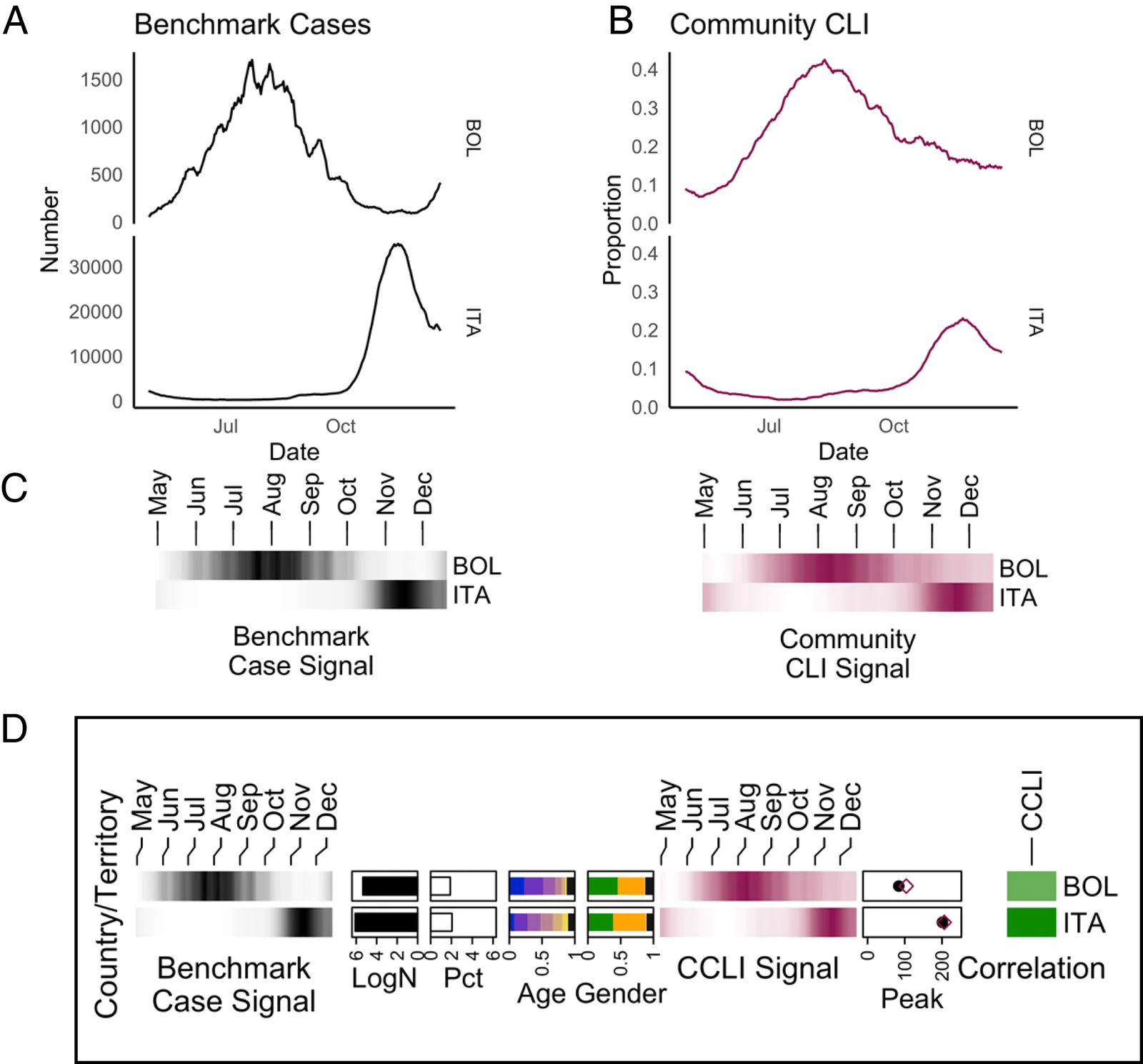
The schematic of COVID-19 case and UMD-CTIS surveillance signal benchmarking globally. For each country and territory, the 7-d smoothed COVID-19 case counts from Our World in Data (*A*) are compared to the survey-weighted CTIS surveillance measure. (*B*) The CCLI signal for Bolivia and Italy is shown for illustrative purposes. The survey-weighted sum of “yes” responses to the surveillance questions (here the CCLI survey question) for each week was divided by the sum of survey weights for all surveys over a 7-d window. (*C*) Time series were normalized to a range of 0 to 1using minimum and maximum during the survey period to allow within- and between-locale comparison of trends across a range of values using color intensity. (*D*) For each country/territory (rows), we combined normalized time series with log_10_ of the number of surveys (black bar chart), percent surveys per population (white bar chart), age and gender distributions (stacked bar charts), peak day (solid black circle, benchmark; open colored shapes, signals) and the benchmark–signal correlation strength (green) in the form of an annotated heatmap.

We sought to determine if we could use a limited set of survey questions about recent symptoms and demographics to predict the individual-level positive test result among respondents who had reported a recent COVID-19 test result (schema in *SI Appendix*, Fig. 3*A*). The goal of this application of surveillance data would be to determine if UMD-CTIS responses can identify the most important symptoms associated with a positive test result. This model might inform isolation of an untested individual or become the basis of a symptom-based surveillance signal for COVID-19 case trends. Such information was needed early in the pandemic and would be valuable should a novel variant or virus cocirculate at any place around the globe. We hypothesized that a basic machine learning model trained on pooled global UMD-CTIS positive and negative test result data—made possible by use of a unified survey instrument—would allow for a rapid collection of symptom profiles of recent positive test result respondents across regions with variable testing practices. The global model performed well on holdout UMD-CTIS data (F1 0.74) and performed better in 77% of countries/territories than models built on local data (*SI Appendix*, Fig. 3*C*). The global model area under the curve of 0.799 was comparable in magnitude to that from a large prospective longitudinal syndromic surveillance data set (11) (Fig. 2*A*). The very high importance of self-reported loss of smell or taste and typical COVID-19 symptoms (e.g., cough, fever) were consistent with prior studies (32, 33) and were generally similar in local models (Fig. 2, and *SI Appendix*, Fig. 3*D*). The young male category was the strongest demographic feature for many local models but was less so in the full global model. We acknowledge the potential for differential testing across locales and within demographic groups, which is a limitation of using data from those tested rather than a “gold standard” prospective study with uniform testing. However, despite these differences in the importance of some model features, the global model outperformed most models trained on local data, in which these very different testing probabilities may limit model building. These initial models for predicting a positive COVID-19 test result could be developed further or could incorporate external data or additional features. Even so, we show it is possible to use a globally deployed surveillance system to bolster a predictive symptom-based model that performs well locally.

Having demonstrated how UMD-CTIS could “crowdsource” a symptom-based prediction model on a global scale, we tested this using a subsample of data from tested respondents either early in their illness or early in the pandemic when initial insights would be valuable (see performance for these and other sensitivity analyses in *SI Appendix*, Fig. 3). The model trained on data from those with a brief illness (0 to 4 d) at the time of the survey performed similarly (F1 0.69) and provided the same top four symptom features for predicting a positive test result (i.e., loss of smell/taste, cough, fever, and chest pain). We also trained an early pandemic model on data from the first 2 wk of the global data (April 27 to May 10, 2020). The early pandemic model performed well (F1 0.74) and identified the four important symptom predictors despite using substantially fewer survey responses for training (*n* = 10,766) than were used to train the full global model (*n* = 407,308). Difficulty breathing was the next most important symptom in the early pandemic model and also increased in importance in longer versus shorter duration of illness models. This variation might reflect syndromic features of those tested early in disease versus those tested early in the pandemic. The ability to pool global data and evaluate early strata further demonstrates the potential utility of a rapidly deployed syndromic surveillance platform to gain early insights applicable to the global community impacted during a pandemic, even when data quality or quantity may otherwise limit the utility of digital surveillance to glean these insights individually in a single country or territory.

### Benchmarking COVID-19 Syndromic Surveillance Signals.

**Fig. 4.**
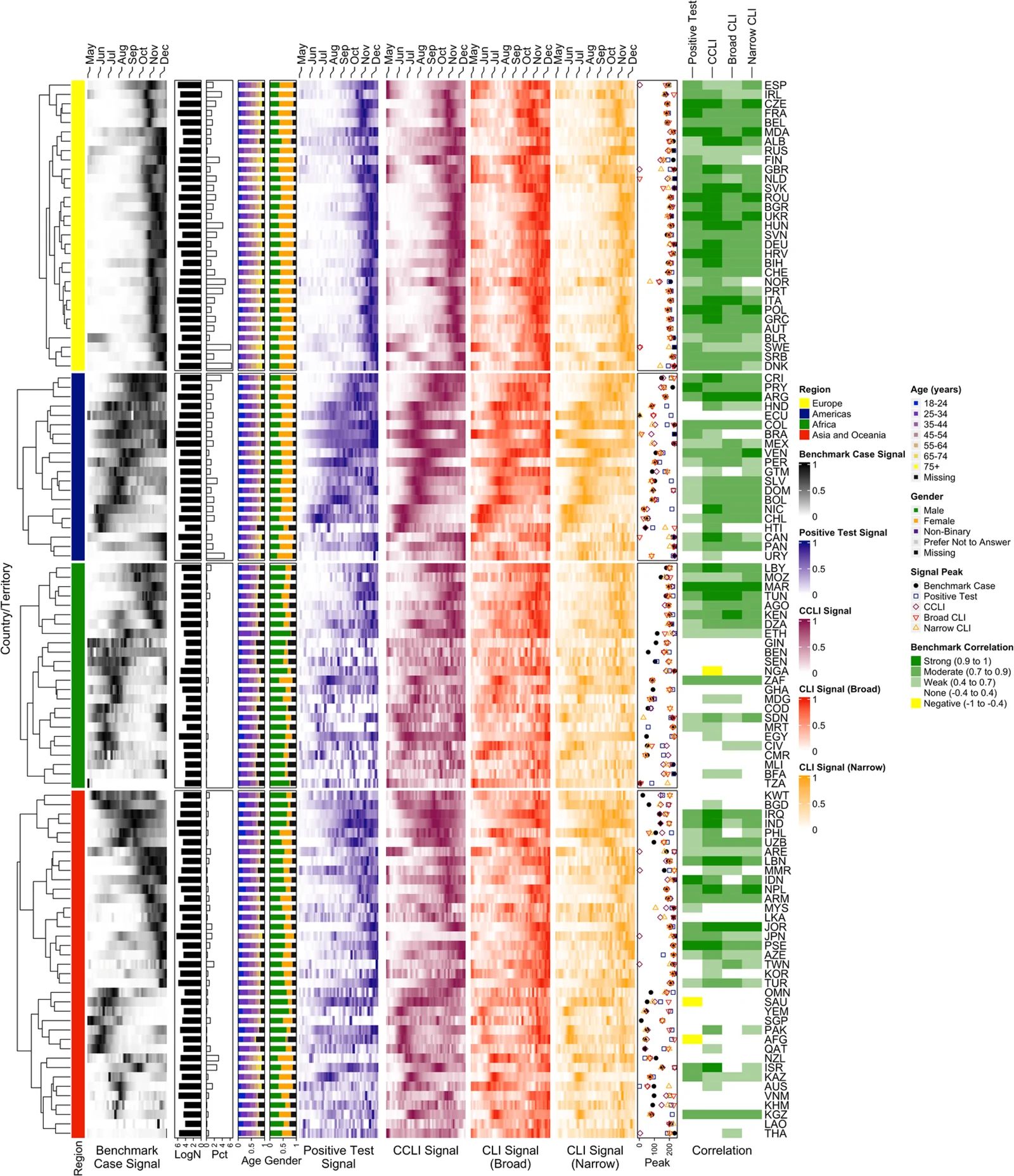
The time series heatmap comparing the benchmark cases to UMD-CTIS–based signals. Refer to the illustration of the generation of the time series heatmap components in Fig. 3. Normalized benchmark (black column) and UMD-CTIS (navy through orange columns) signal time series by country or territory (*Country/Territory*, rows) are clustered by benchmark within geographic regions. Signals include recent positive COVID-19 test result (*Positive Test*), *CCLI*, self-reported fever, cough or loss of smell/taste (*Broad CLI*), or self-reported loss of smell/taste of less than 14 d duration (*Narrow CLI*). The days to peak for each signal is compared (*Peak*) with the benchmark. The Spearman correlation strength (*Correlation*) of UMD-CTIS with the benchmark. Log_10_ surveys (*LogN*) and surveys per population (*Pct*) as bar charts and proportion of surveys for each *Age* or *Gender* as stacked bar charts.

As UMD-CTIS respondent demographics were consistent within many countries during the study period, we hypothesized we could use UMD-CTIS to develop COVID-19 surveillance signals that would function across a range of countries/territories. First, we evaluated the recent positive test result signal, reasoning this would be a suitable comparison for new cases given a positive test result is often used to define COVID-19 cases. As a comparison, we devised two syndromic surveillance signals based on classic COVID-19 symptoms (32, 33), including broad (i.e., loss of smell/taste, cough, or fever) and narrow (i.e., loss of smell/taste with illness < 14 d duration) COVID-like illness (CLI). Lastly, we sought to identify whether human social sensing—that is, using a proxy response of knowing someone with CLI in the respondents’ local community (CCLI)—could provide meaningful trends across the countries/territories covered by the platform, even in areas with lower survey coverage (24). Additionally, this signal was of interest because it could potentially give insights about a broader slice of a population than the population from which survey respondents were sampled (e.g., a health-conscious Facebook user respondent can report on CLI among their less health-conscious, non-Facebook community members) and required a single response from study participants. The signals were purposely derived from separate questions found in different sections of the survey to avoid aberrant responses (e.g., ticking “yes” to all symptoms or entering implausible illness duration) or missingness (e.g., leaving the survey before completion) in one question driving all signals. As illustrated in Fig. 3, benchmark (34, 35) COVID-19 cases per population (black) and UMD-CTIS responses per survey over a 7-d moving window (here CCLI in dark pink) were normalized to allow for visual comparison of epidemic curves versus UMD-CTIS signals across regions and a wide range of values. Overall, the four UMD-CTIS signals reveal the global pandemic “fingerprint” of benchmark case trajectories (Fig. 4).

To facilitate qualitative comparisons of these four signals in their ability to trend benchmark cases, across countries/territories with asynchronous COVID-19 waves and varied survey coverage, we calculated simplified measures to characterize similarity: difference in peak day (φ = signal − benchmark) and Spearman correlation strength (ρ). These measures should be taken as descriptive only. Statistical inference on these time series is possible, but detailed analyses were beyond the scope of this paper and should be tailored to the specific country/territory, survey period, and application of interest. The |φ| may be large if there are multiple waves during the study period. The ρ may be large with nonstationary time series (such as epidemic curves here), even when the time series are not meaningfully associated. Noisy signals not reflective of an epidemic curve (e.g., white noise) might have large |φ| and ρ near zero. Signals with these features would be less informative for surveillance. We ranked signal–benchmark similarity measures within countries/territories (relative to the same benchmark curve) in addition to comparing signal similarity measures between countries/territories.

Overall, UMD-CTIS signals benchmarked well for many signals in many countries and territories (shown graphically in Fig. 4, summarized in *SI Appendix*, Fig. 4). Narrow CLI had the earliest peak relative to benchmark data (median rank φ = 2 [tied with CCLI], median φ = −4.5 d), while CCLI was the most strongly correlated with benchmark data (median rank ρ = 1, median ρ = 0.76). Positive test performed the least well of the four signals by median rank and similarity measure (median φ = ±12 d, median ρ = 0.63). We reasoned that a predicted test positive test signal based on reported symptoms and the global positive test prediction model applied to all UMD-CTIS responses (regardless of testing) might identify benchmark case trends better than reported positive tests in regions with limited UMD-CTIS test reports. The predicted positive test signal ranked better than the reported positive test signal for both similarity measures (median φ = ±7.5, median ρ = 0.66) but did not benchmark better than the other signals. It is presently not possible to distinguish poor benchmarking because of UMD-CTIS signal performance (e.g., biased survey sample) versus lower quality benchmark data in a specific locale (e.g., inadequate reporting and insufficient testing) versus the above caveats. The value of the signals as measures of local disease burden in the absence of adequate benchmark data were not evaluated.

Finally, we filtered for country/territory signals that might be especially useful in syndromic surveillance. We tabulated those that met stringent thresholds for indicating peak timing (−14 ≤ φ < = 7 d) and being at least moderately correlated (0.7 ≤ ρ) with benchmark cases. About half (*n* = 60) of the countries/territories had at least one signal that met the stringent thresholds for these syndromic surveillance signals, while the CCLI signal met the thresholds for the largest number of countries/territories (*n* = 37; 33%). It is possible that combining signals (e.g., CCLI with case takeoff, CLI near peak), including additional survey items (e.g., number CCLI), and incorporating rate of change of the signal could theoretically enhance signals but was beyond the scope of the descriptive analyses presented here. These findings do highlight that very few survey questions, human social sensing, and sampling from the FAUB can nevertheless provide a global data stream to remotely track public health measures locally.

## Discussion

While many areas of the world have access to sophisticated, timely, and reliable methods for tracking COVID-19 impacts over time, there are billions of people living in regions without these resources (36). However, a necessary step in dampening global COVID-19 transmission is the ability to efficiently and quickly monitor events and responses locally. This is especially important as governments downsize disease surveillance programs (37) despite inadequate testing of symptomatic people. Furthermore, there is a constant threat of the evolution of COVID-19 variants, reemergence of seasonal pathogens, and emergence of future pandemic pathogens (38). The UMD-CTIS online survey platform—delivered to social media users, leveraging Facebook-provided infrastructure, and bolstered by public health partners and survey methodologists—has the potential to fill this surveillance gap.

Here, we show syndromic surveillance signals from cross-sectional surveys offered daily to a statistical sample of Facebook users are consistent with benchmark COVID-19 case time series. The UMD-CTIS platform samples from over 2 billion individuals in the FAUB sampling frame, including many in emerging economies (39), and provides survey weights. Despite these advantages, UMD-CTIS may suffer from biases (40) similar to other web surveys when generalizing to the full population, particularly where internet access varies by regional or demographic factors or is related to the outcome of interest. The validity of the test positive signal in relation to benchmark cases demonstrates UMD-CTIS can deliver survey-weighted responses from a meaningful subsample of the test-positive cases in a country/territory over time. Indeed, for many countries and territories, UMD-CTIS respondent demographics are consistent from week to week, and respondent demographics are similar to the census. Taken together, these findings provide good evidence UMD-CTIS can track relevant public health trends and may be more reliable for certain measures than those often used in opt-in surveillance without a clear sampling frame.

The surveillance signal most consistent with benchmark case data was the answer to a single question: “Do you personally know anyone in your local community who is sick with a fever and at least one other symptom?” This simple signal, created as a human social sensor (24), performs in regions with lower survey coverage, effectively scaling up the sample size through respondents reporting CLI on behalf of their local community. This syndromic surveillance signal does not require the respondent to have been sampled during their own, possibly brief, symptomatic period, nor does it require testing infrastructure and capacity. Furthermore, this signal does not require the respondents to have a representative risk profile but rather to be connected to, and thus represent, their local community’s risk profile. This proof of concept has been demonstrated in other settings (41). In contrast, more specific symptom-based signals may be more relevant at higher COVID-19 incidence or when transmission is falling. For example, narrow CLI ranked the best for identifying the epidemic peak. Further studies could evaluate how these signals perform in the absence of benchmark cases (e.g., benchmark case data flat and CCLI signal surge), but this was beyond the scope of this analysis.

Syndromic surveillance relies on syndrome definitions such as CLI and CCLI to derive signals informative of disease activity (42). Early surveillance-based models can rapidly identify relevant symptoms of interest, and it is expected that the symptoms for tracking transmission and guiding self-isolation may evolve over time. Having shown UMD-CTIS respondents who reported having tested positive provide a potentially informative subsample of benchmark cases (and, possibly, their respective syndromes), we predicted positive results using respondent symptoms and demographics in a simple machine learning model. While this model performed well when tested on holdout data, we acknowledge the model is only a starting point. The test result data were drawn from selected populations (i.e., among those tested and reporting in UMD-CTIS) and were pooled across locales with variable testing access. However, despite these limitations, the global model performed better than most individual country/territory models. Training on pooled global data identified the classic CLI symptoms (e.g., self-reported loss of smell or taste, cough, fever, and chest pain) (10, 11, 25, 33) even when limiting data to the first 2 wk of the study period. A symptom-based syndromic signal developed from this predictive model performed better than the reported positive test signal. Thus, we demonstrate it is feasible to use a “crowdsourced” global model to enhance local syndromic surveillance. UMD-CTIS enables the rapid identification of predictive symptoms and the development of a syndromic signal, both of which can be applied to countries/territories even when and where UMD-CTIS reported tests results in these locales might be sparse.

Remote syndromic surveillance platforms, including UMD-CTIS, may have confounding, selection, and measurement biases. Generally, health-engaged subjects participate and continue to participate over time, data are self-reported, digital interface access is necessary, and outbreaks may skew epidemiological parameter estimates (29, 43, 44). Designed as a repeated cross-sectional survey without linkage of repeated user responses, UMD-CTIS cannot directly evaluate individual-level effects or disease trajectories. Ecological bias must be considered; though, in this study, the outcome of interest is primarily population-level trends, not individual-level causal effects. The FAUB sampling frame is large, and through the survey weights, it adjusts for the known probability of selection into the analytic sample. We show, even in locales with age–gender skewed samples, that UMD-CTIS signals rise and fall similarly to local case trajectories. However, the powerful and efficient strategy of combining user-based sampling of a defined cohort and survey weights to improve representativeness is unique among existing syndromic surveillance tools.

We attempted to and were able to benchmark four signals derived from different questions and question types with minimal data manipulations. Additional important considerations are the relatively high baseline of many metrics, perhaps because of nonresponse patterns, low-quality response patterns because of distracted respondents, poor internet quality, or so-called “trolling” behavior. Survey response biases for individual questions did not appear to obscure time trends; locales that benchmarked well for one more often benchmarked well for them all. The four signals were generally equally noisy in the locales that benchmarked poorly. We observed some demographic shifts away from younger and male respondents over time to a lesser or greater extent across all locales despite variable COVID-19 waves and other factors. Thus, rising and falling locale-specific signals are not likely from the ebb and flow of nonresponse patterns. In fact, over the course of a wave of a few months’ duration, the demographic shifts are relatively small for most locales. Thus, we showcase that a strength of this survey strategy is in time trends of syndromic surveillance signals at a global scale (22). We acknowledge that there can be trend breaks with survey versions (e.g., questions on testing were fielded to those symptomatic initially and then to all respondents), though these can be mitigated with sensitivity analyses (e.g., stratified by version), nonparametric correlations methods of trends rather than the use of the specific point estimates (as we implemented in this analysis), and benchmarking of other demographic features to track changes in representativeness. Other outputs such as estimation of absolute burden, cumulative incidence of an event, or *R_t_* would require methods to deconvolute cross-sectional responses and adjust for the baseline response rate.

The purpose of the UMD-CTIS is not to provide exact and unbiased on-time point estimates but instead to allow for the analysis of time trends. Despite acknowledged limitations, the potential value of the UMD-CTIS is substantial. Compared to the Gallup World Poll (45), one of the largest global surveys, the UMD-CTIS survey is conducted on a daily (not annual) basis by leveraging a social-media–based instrument instead of telephone and in-person interviews (46). Gallup World Poll interviews about 1,000 subjects per country per year and has with its tested methodology a higher chance of unbiased point estimates. UMD-CTIS, in contrast, sampled 5 to 10 times that per country or territory per week and is better suited for measurements that change quickly over time. In addition, it is lean on local infrastructure and human resources. UMD-CTIS is the largest ongoing real-time, remote, global health survey ever conducted.

Given its rapid sampling mechanism, the UMD-CTIS survey can adapt to on-demand public health needs and provide insights for a large segment of the global population. With each survey version, new questions have been added in conjunction with epidemiologists, local government input, and research partners. Other studies have used these data to investigate a range of insights, including masking effectiveness, testing practices, and vaccine hesitancy, and there is potential to further understand knowledge, attitudes, and practices through this high-density data stream (30, 47–50). Indeed, the survey has most recently incorporated items to survey social impacts, vaccine uptake, and vaccine hesitancy (31). With the expected return of seasonal respiratory infections (51) and the possibility that the influenza burden may be much more severe (52), as well as the possible emergence of other known or novel pathogens of significance, the sampling strategy developed for this global health tool may be invaluable, especially in conjunction with government reporting, viral surveillance, and region-specific syndromic surveillance methods (21, 23). For example, UMD-CTIS could rapidly deploy new survey questions to collect respondent test results for a novel variant or virus, where and when these tests become available and accessible, or to track an emerging “community viral-like illness” as the syndromic definition evolves. In conclusion, UMD-CTIS has shown to be a valid and powerful means of monitoring waxing and waning impacts of COVID-19 in a range of settings including underrepresented locales.

## Materials and Methods

### UMD-CTIS Survey.

This study was approved by the Boston Children Hospital Institutional Review Board (IRB, P00023700). This research is based on survey results from UMD-CTIS, approved by the UMD IRB (1587016-10) and described previously (22). Briefly, each day, starting on April 23, 2020, a subset of the 2.6 billion-person global FAUB was sampled and invited to participate via a special banner on the top of their news feed (summarized in Fig. 1*A*). Both respondents and nonrespondents might be sampled and invited again to take the survey (a few weeks or months after the initial request, depending on the population density of their area). Designed as a repeated cross-sectional survey, there is no linkage of responses of resampled users in the UMD-CTIS data. Facebook users were pushed to a web-survey platform utilizing a token system. UMD’s Qualtrics license was used to collect the web survey data. All respondents must self-report age ≥18 y and provide consent before participating in the survey. All survey items were collected by UMD using a web-based questionnaire translated into commonly spoken languages within each of the 250 countries and territories surveyed. The survey instrument was updated periodically with input from survey methodologists, epidemiologists, and public health specialists to address timely public health questions (e.g., masking behaviors and vaccine hesitancy). UMD-CTIS survey data span inception through the present and include responses from 12 survey versions. UMD compiled daily, real-time microdata, such as those used in this study, as well as publicly available survey-weighted aggregated data (25).

### Survey Weights.

Facebook provided UMD with survey weights for respondents in 114 of the 250 countries and territories. The weighting methodology has been described in greater detail previously (53). Briefly, survey weights adjust for sampling bias, nonresponse bias, and country/territory-level demographics from the United Nations Population Division 2019 World Population Projections. The weights adjust for known sampling bias in the form of base weights and nonresponse bias in the form of inverse propensity score weighting (IPSW). There are 71 locales where the weights are ranked using age-by-gender and administrative level-1 region population totals as well as 43 where the weights are poststratified using age-by-gender only. However, demographic data reported to UMD via the Qualtrics web survey may differ from the demographic data derived from respondents’ Facebook user profiles that were used to develop base weights that adjust for sampling biases. Furthermore, the weights may not account for coverage biases. For example, the Facebook population may differ from the country or territory population on more features than just age, gender, or administrative level-1 region, and/or the IPSW nonresponse model may suffer from omitted variable bias.

### Study Design.

This study uses UMD-CTIS microdata from the 114 countries and territories with survey weights from the first 34 full ISO weeks from UMD-CTIS inception through just before COVID-19 vaccine rollout (weeks 18 to 51, April 27, 2020, to December 20, 2020). Surveys for versions 1 through 6 that were in use during this study period are available online (25) and are summarized in *SI Appendix*, Table 1. The data were accessed on January 21, 2021. All analyses are retrospective using self-reported cross-sectional survey responses and the provided survey weights. We compared UMD-CTIS demographics to census demographics over countries/territories and time, developed a basic machine learning model for predicting COVID-19 positive test result from symptoms and demographics, and benchmarked UMD-CTIS signals to COVID-19 cases.

### Demographic Distributions and Consistency.

The number of UMD-CTIS surveys per capita (34) was compared. For each locale (i.e., country or territory) and locale week, we compared UMD-CTIS respondents to census demographics. First, we computed the proportion (*P_g,_*_UMD-CTIS_) of UMD-CTIS respondents in demographic groups (*g*) defined by gender (male and female) and age (young, 18 to 34; middle, 35 to 54; and elderly, ≥55 y) for each country/territory. The denominator was the sum of all surveys in the six age–gender groups for that locale. Surveys with missing demographic data or nonbinary gender were not included. We then computed the proportion (*P_g,_*_Census_) of the census population in each group (27). Age bins for census data were consistent with those in the UMD-CTIS survey response options except that the young age corresponded to the 20- to 34-y census age category. The denominator of the census proportion was the sum of the population in all six age–gender groups for that locale. The population outside of these age ranges or with nonbinary gender was not included. For each locale, we calculated the difference in the proportion for that age–gender group in UMD-CTIS versus census (*D_g_* = *P_g,_*_UMD-CTIS_ − *P_g,_*_Census_). For each locale, the mean absolute difference (δ) across all groups was calculated by taking the average of the absolute value of the age–gender differences for the six groups (δ = *Σ*_*g*_ |*D_g_*|/6). We specified that <0.1 difference would be a reasonable variation based on previously published data on demographic skew in a preexisting internet-based syndromic surveillance platform (29). To evaluate for variation in demographic distributions over the study period, we calculated the above two measures for each week (*w*) and locale (i.e., *D_g,w_* and δ*_w_*). For each locale, week, and measure, we then calculated the difference of the locale–week measure versus the median [i.e., *Δ*_X,_*_w_* = *X_w_* – median(*X_w_*)], giving *Δ*_*Dg,w*_ and *Δ*_δ*,w*_ for the change in the weekly difference of proportion measures and the change in the weekly mean absolute difference measures, respectively.

### Prediction of Global and Local COVID-19–Positive Test.

To enable evaluation of COVID-19 case trends with minimal testing data, we conducted a retrospective diagnostic classification analysis by training a gradient-boosting decision tree binary classifier. Four base-modeling methods (Logistic Regression, Gaussian Naive Bayes, Support Vector Machine, and Light Gradient Boosting Machine) were compared to identify the Light Gradient Boosting Machine (LightGBM) as the highest performing at a moderate time cost. Therefore, this method was used for all analyses and is presented throughout (LightGBM in Python 3.8 with Shapley values visualization package for predictor visualization). Since we aimed to build a reasonable model with a limited feature set, the input features were self-reported age–gender categories (four using demographic groups noted above (*Demographics Distributions and Consistency*) but collapsing the middle to elderly ages and creating a fifth strata with missing age, nonbinary gender, or missing gender) and responses to 12 symptoms asked of survey participants in versions 1 through 6. Symptoms without responses were assumed to be “no” if at least one symptom response was provided. The data used for model building and analytical evaluation were obtained by filtering the surveys containing these selected data fields and splitting them into training and holdout sets as shown in *SI Appendix*, Fig. 3*A*. When preparing the two sets, each of the five covariate-specific strata for each locale was split 3:1. Since the test negative and positive data were inherently imbalanced (median [IQR] negative/positive 4 [2, 10]) and to limit overfitting, the training set was down- or up-sampled to ensure balanced classes before 10-fold stratified cross-validation. Stratified cross-validation ensured training and test sets across each cross-validation fold had approximately matched proportion of positive and negative classes. The outcome variable was the self-reported positive versus negative recent COVID-19 test result (missing responses excluded). Models were trained on each locale and a pooled global data set applied to unweighted training data with balanced classes and 10-fold stratified cross-validation. We then tested the global model on each individual locale and vice versa. See *SI Appendix*, Fig. 3 for secondary and sensitivity analyses using the outcome of testing, excluding early survey versions of the highest F1 countries/territories, or stratification by illness duration or study period. The global model was hyperparameter turned (parameters in *SI Appendix*, Table 2).

### Benchmarking Syndromic Surveillance Signals.

UMD-CTIS trends over time were compared to benchmark data from the publicly available Our World in Data (OWID) case trends (34) accessed April 23, 2021. Survey-weighted (53) proportions were calculated by locale and day for 7-d windows. Seven-day smoothing was applied to the time series by taking the survey-weighted sum of respondents endorsing the signal criteria (e.g. symptom present) d over the survey-weighted sum of all respondents for the prior week day by day. Survey questions without responses were coded as “not present,” “no,” or “false” except when noted (though some survey versions differentiate missing as seen but not answered versus not seen and not answered). We defined the peak day as the day with the highest signal value. The difference in peak day (φ = signal − benchmark) was used to compare signal to benchmark. Spearman correlations (ρ, corr.test from psych library in R) of OWID benchmark data versus UMD-CTIS signals were calculated and categorized by strength. We compared the top-ranked signals across countries/territories with more negative φ and higher ρ, giving higher ranks for these similarity measures. We also compared the median similarity measure between signals. To identify optimal syndromic surveillance signals for each country/territory, we specified a stringent threshold of −14 d ≤ φ ≤ 7 d and 0.7 ≤ ρ. We reasoned that lag times of 0 to 7 d could still be worthwhile in regions with some benchmark case reporting delays (we do not consider severe delays or potentially absent benchmark case data), that lead times more than 14 d might be from time series with multiple peaks (i.e., not informative), and that lower ρ would filter out noisier signals. The primary UMD-CTIS signals were recent positive test results reported per survey (i.e., positive test), CCLI, broad CLI (i.e., loss of smell/taste, cough, or fever in prior 24 h), and narrow CLI (loss of smell/taste and illness duration of <14 d). For trend visualization, smoothed daily country/territory trends (*X_c,t_*) were normalized to range from 0 to 1 using the minimum and maximum over the study period (*T*) for each locale [i.e., *Y_c,t_* = (*X_c,t_* − min(*X_c,t_* over all *T*)/max(*X_c,t_* − min(*X_c,t_* over all *T*) over all *T*))] and were grouped into four geographic regions. Benchmark data were used for clustering in the visualization (using row clustering method=”complete” in Heatmap from ComplexHeatmap 2.3.4, R 3.6.3, https://www.R-project.org/) within geographic regions of the world.

## Supplementary Material

 Appendix 01 (PDF)

## Data Availability

Access to the CTIS data can be can be requested from Facebook Data for Good website (https://dataforgood.facebook.com/dfg/docs/covid-19-trends-and-impact-survey-request-for-data-access). The UMD Global CTIS Open Data API, Microdata Repository, and contingency tables are available from The University of Maryland Social Data Science Center Global COVID-19 Trends and Impact Survey website (https://covidmap.umd.edu). Protocols, code, and materials are available via GitHub (https://github.com/BCH-IDHA/CTIS).
