# Supplementary material for: Global monitoring of the impact of the COVID-19 pandemic through online surveys sampled from the Facebook user base
Source: Proc Natl Acad Sci U S A. 2021 Dec 13;118(51):e2111455118. doi: 10.1073/pnas.2111455118 (PMC8713788; doi:10.1073/pnas.2111455118)
Supplement: Supplementary file 1 [file pnas.2111455118.sapp.pdf]

PNAS
www.pnas.org

Supplementary Information for

Global Monitoring of the Impact of the COVID-19 Pandemic through Online Surveys Sampled from the Facebook User Base

Christina M. Astley[a,b,c,d,1], Gaurav Tuli[b,1], Kimberly A. Mc Cord[b,1], Emily L. Cohn[b], Benjamin Rader[b,e], Tanner J. Varrelman[b], Samantha L. Chiu[f], Xiaoyi Deng[f], Kathleen Stewart[g], Tamer H. Farag[h], Kris M. Barkume[h], Sarah LaRocca[h], Katherine A. Morris[h], Frauke Kreuter[f,i,3], John S. Brownstein[b,c,3]

[a] Division of Endocrinology, Boston Children's Hospital, Boston, MA 02115;
[b] Computational Epidemiology Lab, Boston Children's Hospital, Boston, MA 02115;
[c] Harvard Medical School, Boston, MA 02115;
[d] Broad Institute of Harvard and MIT, Cambridge, MA 02142;
[e] Department of Epidemiology, Boston University, Boston, MA 02118;
[f] Joint Program in Survey Methodology, University of Maryland, College Park, MD 20742;
[g] Center for Geospatial Information Science, University of Maryland, College Park, MD 20742;
[h] Meta, Menlo Park, CA 94025; and
[i] Department of Statistics, Ludwig-Maximilians-Universität, Munich 80539, Germany

[1] C.M.A., G.T., and K.A. Mc Cord contributed equally to this work.
[2] To whom correspondence may be addressed. Email: christina.astley@childrens.harvard.edu.
[3] F.K. and J.S.B. contributed equally to this work.

This PDF file includes:

Supplementary Figures 1 to 4
Supplementary Tables 1 to 2

Supplementary Figure 1: UMD-CTIS coverage globally. Surveys per population (orange) during the study period (April 27, 2020 - December 20, 2020) in relation to COVID-19-related biomedical citations per population (blue) for the N=144 countries and territories with survey weights. Locales not sampled by UMD-CTIS or without survey weights are shown in grey. Surveys per population was moderate to high for much of Europe, and Central and South America, and low for much of Africa and Asia. Carnegie Mellon University in collaboration with Facebook oversees a similar, but not identical, study (CMU-CTIS) in the United States. The coverage of this syndromic surveillance platform spans a broad range of locales globally. (a) The survey serves many locales that have been relatively under-represented in COVID-19 biomedical literature to date. Citation counts pulled from Pubmed, Medxriv and Bioxriv on July 7, 2021 using the search logic ("COVID-19" OR "SARS-CoV-2" OR "2019-nCoV" OR "ncov2019") AND (Locale Name OR Alternative Locale Name). Biomedical citations are a very rough proxy for COVID-19-related research, and are not equivalent to availability of accurate and timely public health data in a country. (b) We also show the survey coverage in relation to Health Development Index (HDI, Our World In Data. https://ourworldindata.org/ Accessed October 7, 2021). Importantly, the platform requires no logistical or financial support on the part of local public health officials. For those locales for which additional syndromic surveillance data would be beneficial, UMD-CTIS should be considered and evaluated in the context of that specific locale's needs at that time.

(a) UMD-CTIS coverage in relation to biomedical literature coverage

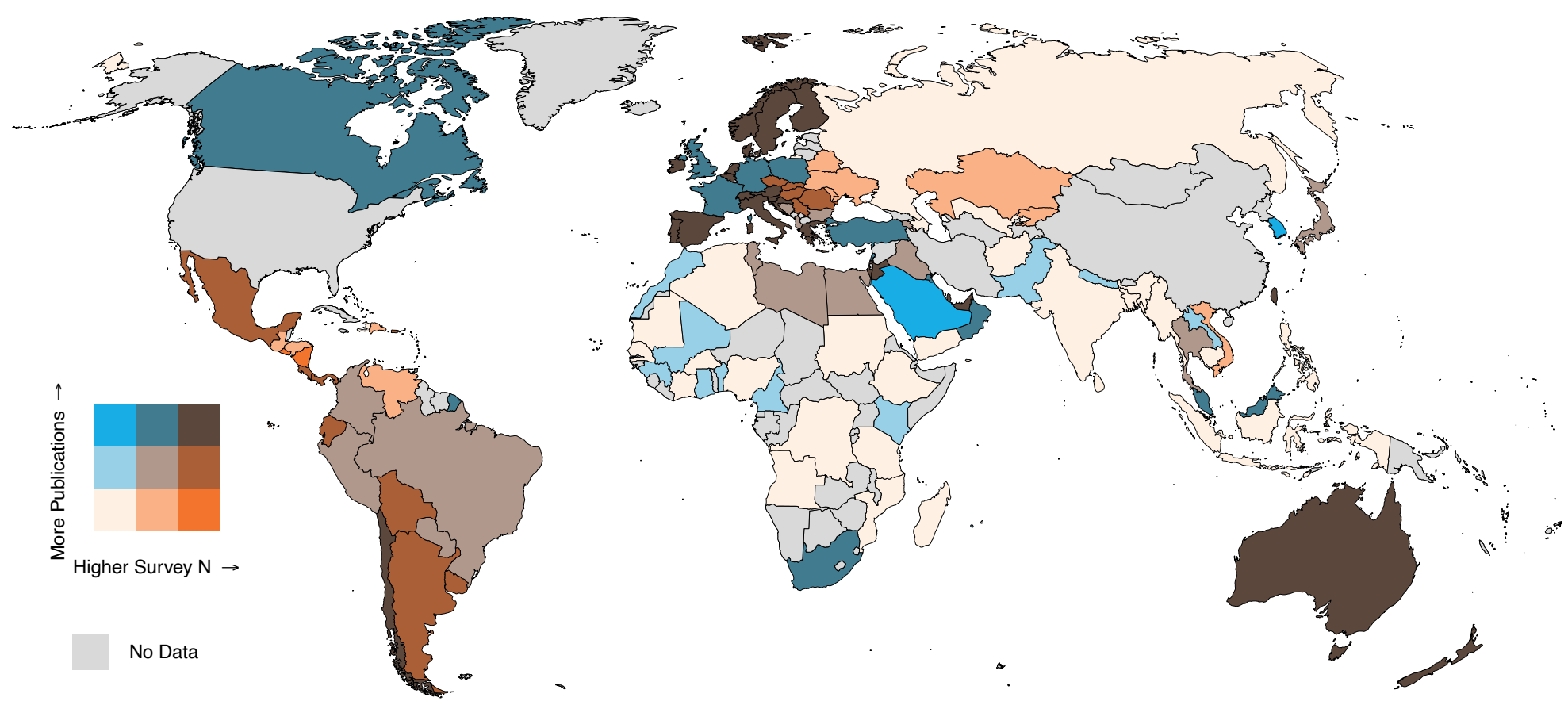

(b) UMD-CTIS coverage in relation to HDI

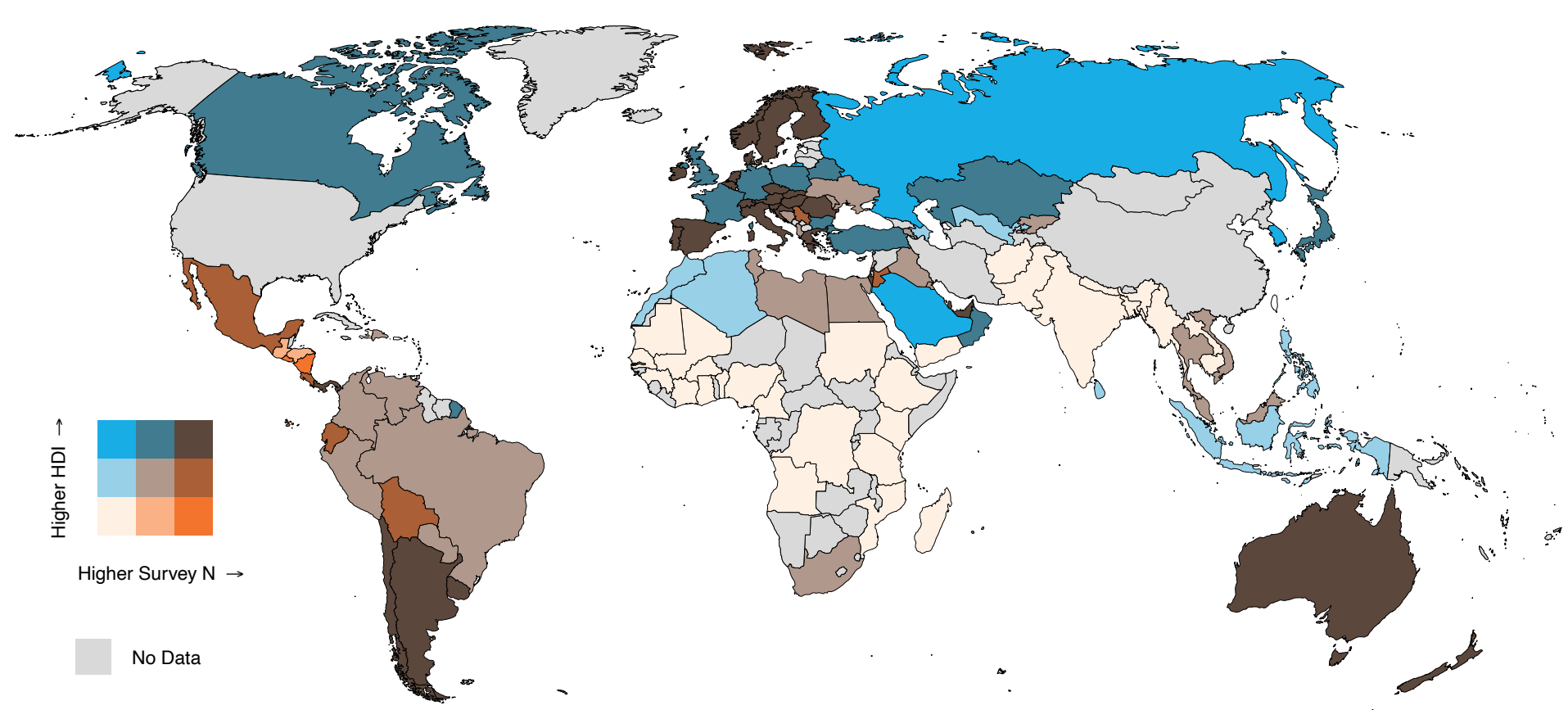

Supplementary Figure 2: Within-locale variation in mean absolute differences from census over the study period. Within-locale variation in mean absolute differences across age-gender groups, by week, relative to the locale median across weeks (i.e. $\Delta_{\delta,w} = \delta_w - \text{median}(\delta_w)$, where $\delta_w = \Sigma_g |D_{g,w}|/6$ for all age-gender groups g).

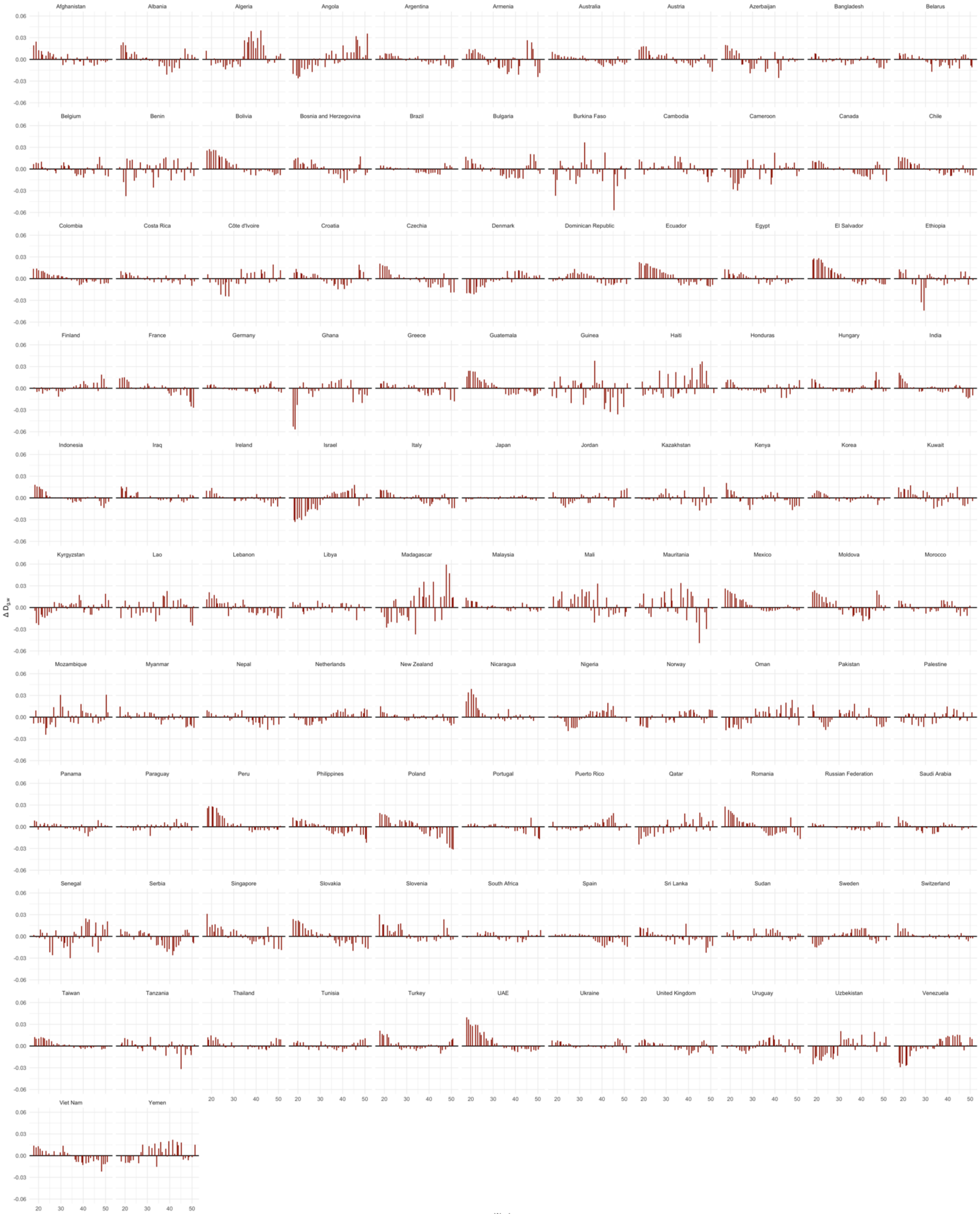

Supplementary Figure 3. Machine learning models to predict individual positive test result.

(a) Schematic diagram of the data flow for the global model. Diagram showing the full sample, and subsampling required to obtain a labelled data set for machine learning using symptoms and basic demographic factors. Down-sampling of the majority class to limit over-fitting was conducted after splitting the data into testing and validation sets, within each demographic group. After splitting the data, we used basic LightGBM to train and test country/territory- and globe-specific models. We hyperparameter tuned the global model with grid search. There was a 0.7% improvement in F1 with hyperparameter tuning. Subsequent models were not hyperparameter tuned. The global model performance overall F1 (F1 0.74), with performance for positive and negative tests in the cross-validation (0.71, 0.78) and holdout data (0.60, 0.89), respectively. Overall F1 for the holdout are presented subsequently. We took a similar approach for predicting receiving a test in the global data set. Not surprisingly, the prediction of recently tested performed worse (F1 0.59) than positive test result. This suggests that symptoms in the prior 24 hours and demographic features were less relevant, and there are other key predictors of receiving a test (e.g. spatiotemporal access, regulatory variability, contact history, high-density housing or neighborhood, and profession). These were not included the model, and further evaluation was beyond the scope of this study. **(b and c) Sensitivity analysis of model performance for individual country/territory models.** We compared the global model performance by applying the global model to holdout country/territory data, and country/territory models to their respective country/territory holdout data. We also conducted a sensitivity analysis restricted to the countries/territories that did not have the ten highest local model F1 scores. We aimed to test whether these countries were driving model features, perhaps due to unusual response patterns or data distributions. **(b)** There was no substantial change in the performance of the restricted global model (x-axis) on local data compared to the global model (difference on y-axis). Difference in performance is plotted versus performance, by country and territory, along with a regression line indicating limited correlation. **(c)** When comparing the performance of the global model (diamond) to the local (circle) models, for most countries and territories, the global model performed better on local data than the local model. Blue (higher) and red (lower) lines show change in performance when using the global model compared to the country/territory model. Graph is arranged by F1 of the global model applied to country/territory data. Median [IQR] F1 of 0.71 [0.64, 0.75], while the global model tested on local data F1 median [IQR] F1 of 0.72 [0.66, 0.76]. **(d-k) Comparison of model features and performance over time.** A model trained on early pandemic data from the first 2 weeks of the study period performed well (F1=0.74) when tested on holdout data from the full study period (the same holdout set used for the global model). Performance was also similar (F1=0.74) in sensitivity analyses omitting training and holdout data from survey versions 1-4 (prior to June 26, 2020), as these versions asked only symptomatic respondents about test results. We compared models trained and then tested on data limited to strata defined by duration of illness (brief 0-4 days, short 5-9 days, medium 10-14 days, long 15-21 days, after rounding duration down to the nearest integer), reasoning that access to testing throughout the course of illness likely varied. **(d)** Summary of model features and performance. Confusion matrices for **(e)** global, **(f)** drop version 1-4, **(g)** first 2 week, and **(h-k)** duration of illness strata.

(a)

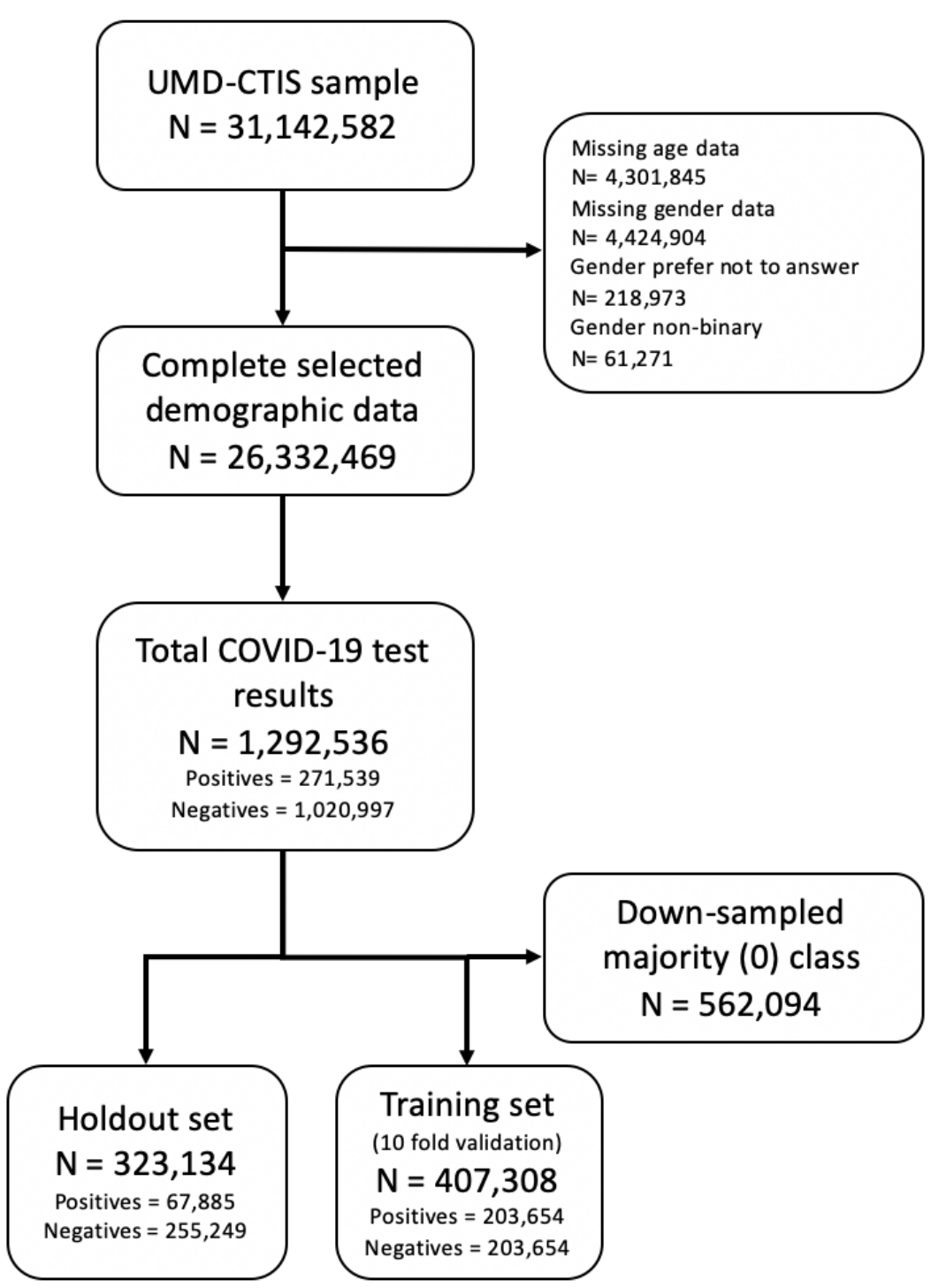

(b)

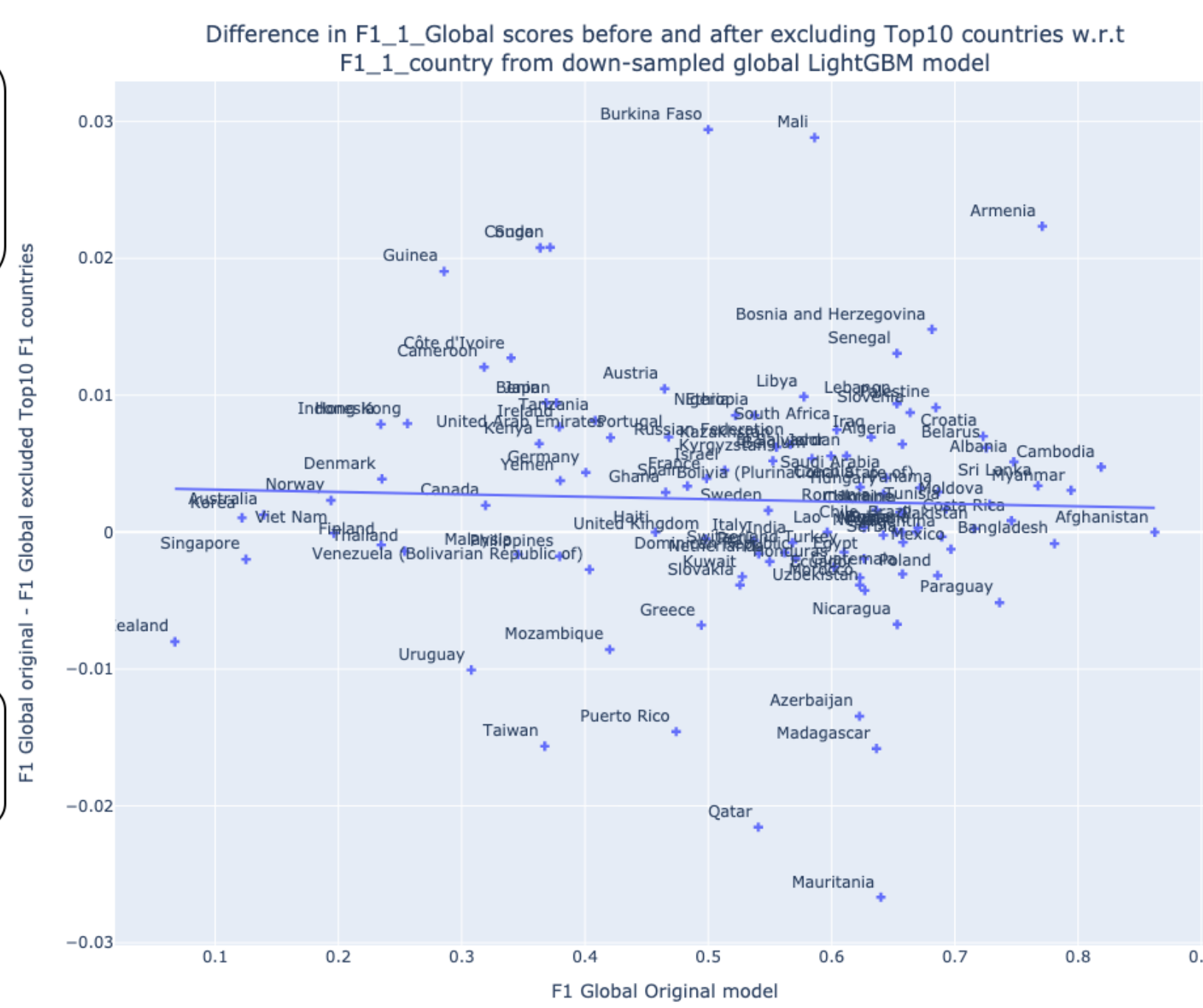

(c) Comparison of local versus global model performance.

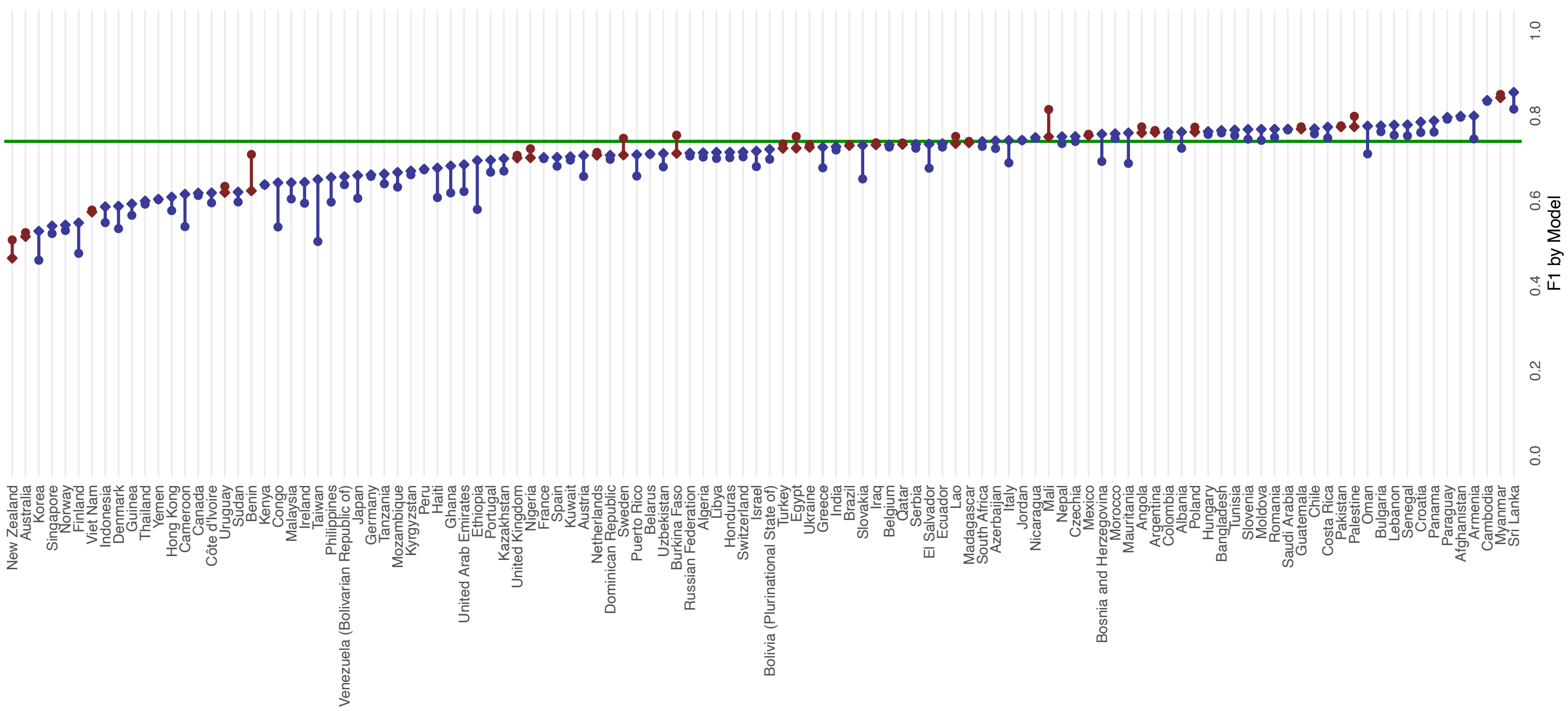

(d) Comparison of global versus illness duration strata, early pandemic and dropped versions 1-4 models

	**Model**						
	**Global**	**Duration of Illness Strata**				**Study Period Strata**	
**Feature**		**Brief 0-4 days**	**Short 5-9 days**	**Medium 10-14 days**	**Long 15-21 days**	**2 week (Early pandemic)**	**Versions 1-4 drop**
Loss smell/taste	(1)	(1)	(1)	(1)	(1)	(1)	(1)
Cough	(2)	(3)	(7)	(6)	(6)	(4)	(2)
Fever	(3)	(2)	(2)	(4)	(2)	(2)	(3)
Chest pain	(4)	(4)	(3)	(2)	(3)	(5)	(4)
Fatigue	(5)	(10)	(6)	(3)	(5)	(11)	(5)
Ache/pain	(6)	(5)	(5)	(9)	(12)	(8)	(6)
Eye pain	(7)	(6)	(4)	(11)	(8)	(13)	(8)
Young male	(8)	(8)	(15)	(13)	(16)	(3)	(9)
Headache	(9)	(7)	(8)	(7)	(7)	(9)	(7)
Diff. breathing	(10)	(13)	(12)	(8)	(10)	(6)	(11)
Old female	(11)	(15)	(14)	(12)	(11)	(15)	(10)
Young female	(12)	(9)	(10)	(10)	(13)	(7)	(12)
Old male	(13)	(16)	(16)	(16)	(15)	(16)	(13)
Runny nose	(14)	(12)	(9)	(5)	(4)	(12)	(14)
Nausea	(15)	(14)	(13)	(15)	(14)	(14)	(15)
Sore throat	(16)	(11)	(11)	(14)	(9)	(10)	(16)
**F1 score**	**0.74**	**0.69**	**0.78**	**0.76**	**0.73**	**0.74**	**0.74**
**Training Set (N)**	**407,308**	**64,046**	**102,054**	**64,100**	**40,884**	**10,766**	**358,774**
**Holdout Set (N)**	**323,134**	**64,157**	**37,930**	**21,629**	**14,070**	**323,134**	**277,966**

(e) Global

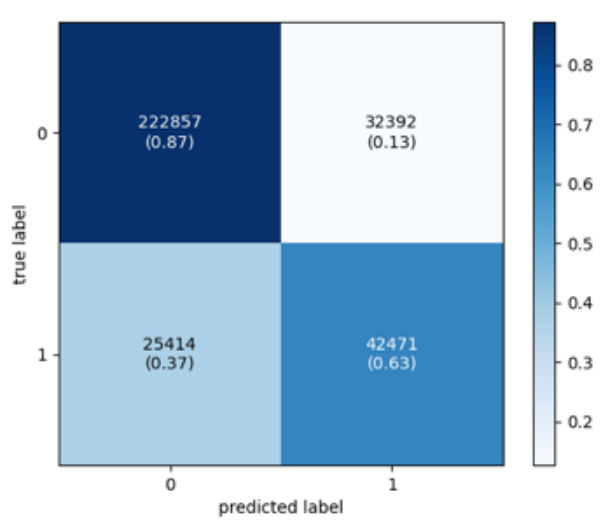

(f) Dropped survey version 1-4

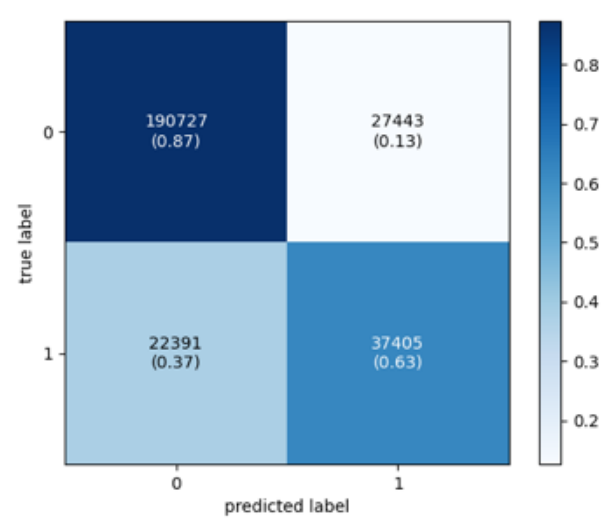

(g) First two-weeks

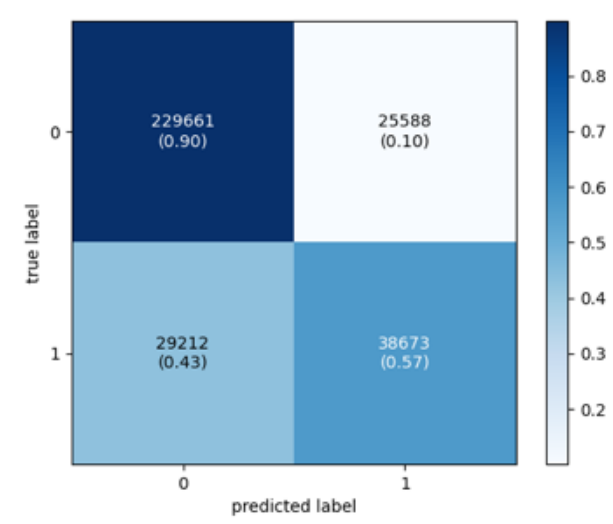

(h) Brief duration (0-4 days)

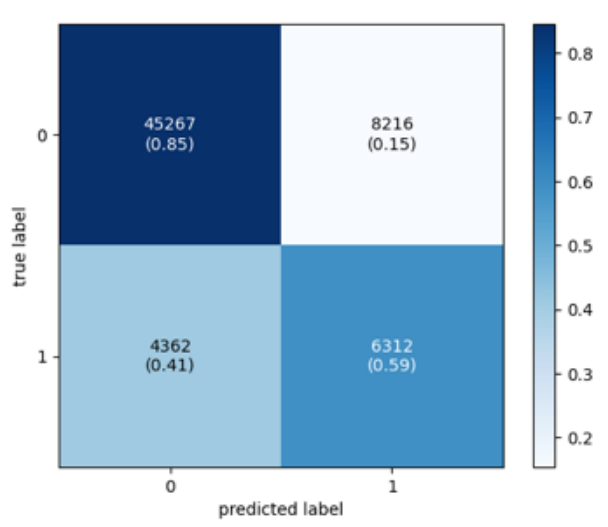

(i) Short duration (5-9 days)

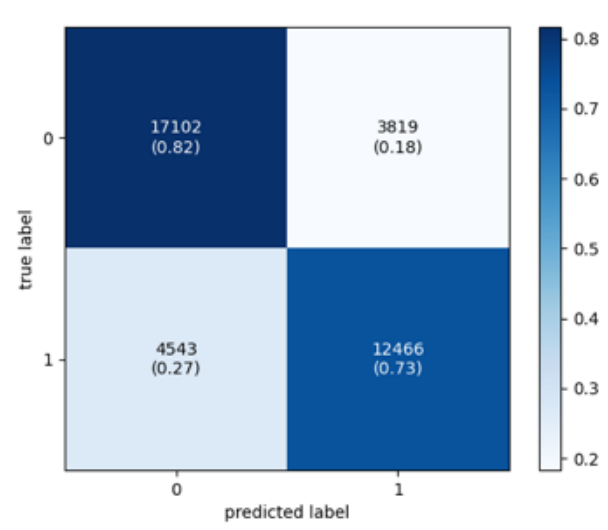

(j) Medium duration (10-14 days)

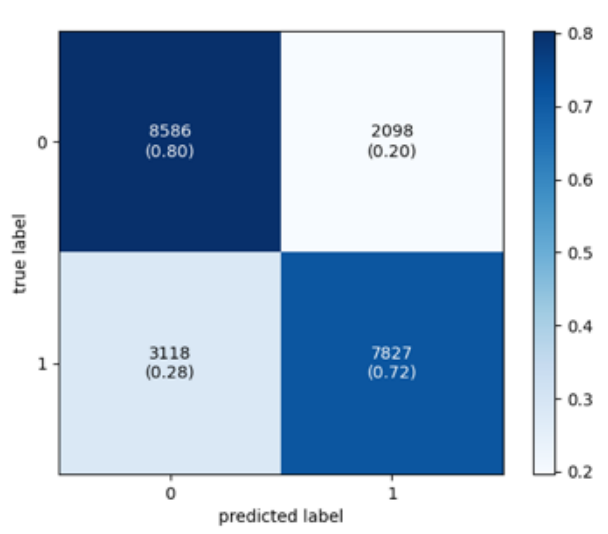

(k) Long (15-21 days)

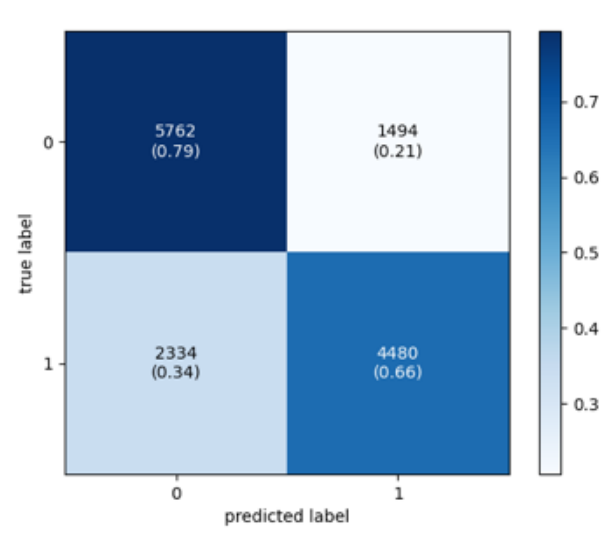

Supplementary Figure 4. Distribution of Spearman Correlations between Benchmark and UMD-CTIS Signals
(a) Distribution summary of Spearman correlations (ρ) and difference in peak day (φ) measures comparing median within-country/territory signal rank, median value of measure by signal, and number of country/territories meeting surveillance thresholds by signal. (b) Distribution summary of correlations (ρ) and absolute difference in peak day (|φ|) within subgroups of the other similarity measure. Median |φ| <= 14 for the groups and signals with moderate to high ρ. Median ρ > 0.7 for groups and signals with |φ| <= 14. (c) Histograms of Spearman correlations for each of four primary signals, with color annotations matching those of Figure 4 in the main text. The median correlations are shown as dotted lines and are reported in the table below. N=112 countries were included in this analysis.

(a)

	**Median rank**		**Median value**		
**Variable**	**Correlation (ρ)**	**Difference Peak Day (φ)**	**Correlation (ρ)**	**Difference Peak Day (φ)**	**Number countries/territories with 0.7 <= ρ & -14 <= φ <= 7**
CCLI	1	2	0.76	1	37
CLI (broad)	3	3	0.66	4	27
CLI (narrow)	3	2	0.67	-4.5	25
Pred. positive test	3	3.5	0.66	7.5	26
Positive test	4	4	0.63	12	20

(b)

	Median |ϕ| by ρ Group and Signal				Median ρ by |ϕ| Group and Signal	
	-1 <= ρ < 0.4	0.4 <= ρ < 0.7	0.7 <= ρ < 0.9	0.9 <= ρ <= 1	|ϕ| <= 14	14 < |ϕ|
Positive test	92.5	17.5	13.0	6.0	0.78	0.47
Predicted positive test	104.0	19.0	10.0	6.5	0.77	0.42
CCLI	80.0	74.0	8.0	8.0	0.87	0.58
CLI (broad)	102.0	44.0	9.0	9.5	0.75	0.50
CLI (narrow)	72.0	15.5	14.0	7.0	0.76	0.42

	N by ρ Group and Sigal				N by |ϕ| Group and Signal	
	-1 <= ρ < 0.4	0.4 <= ρ < 0.7	0.7 <= ρ < 0.9	0.9 <= ρ <= 1	|ϕ| <= 14	14 < |ϕ|
Positive test	38	24	39	11	49	63
Predicted positive test	37	22	45	8	53	59
CCLI	24	22	37	29	52	60
CLI (broad)	36	21	45	10	50	62
CLI (narrow)	37	22	43	10	48	64

(c)

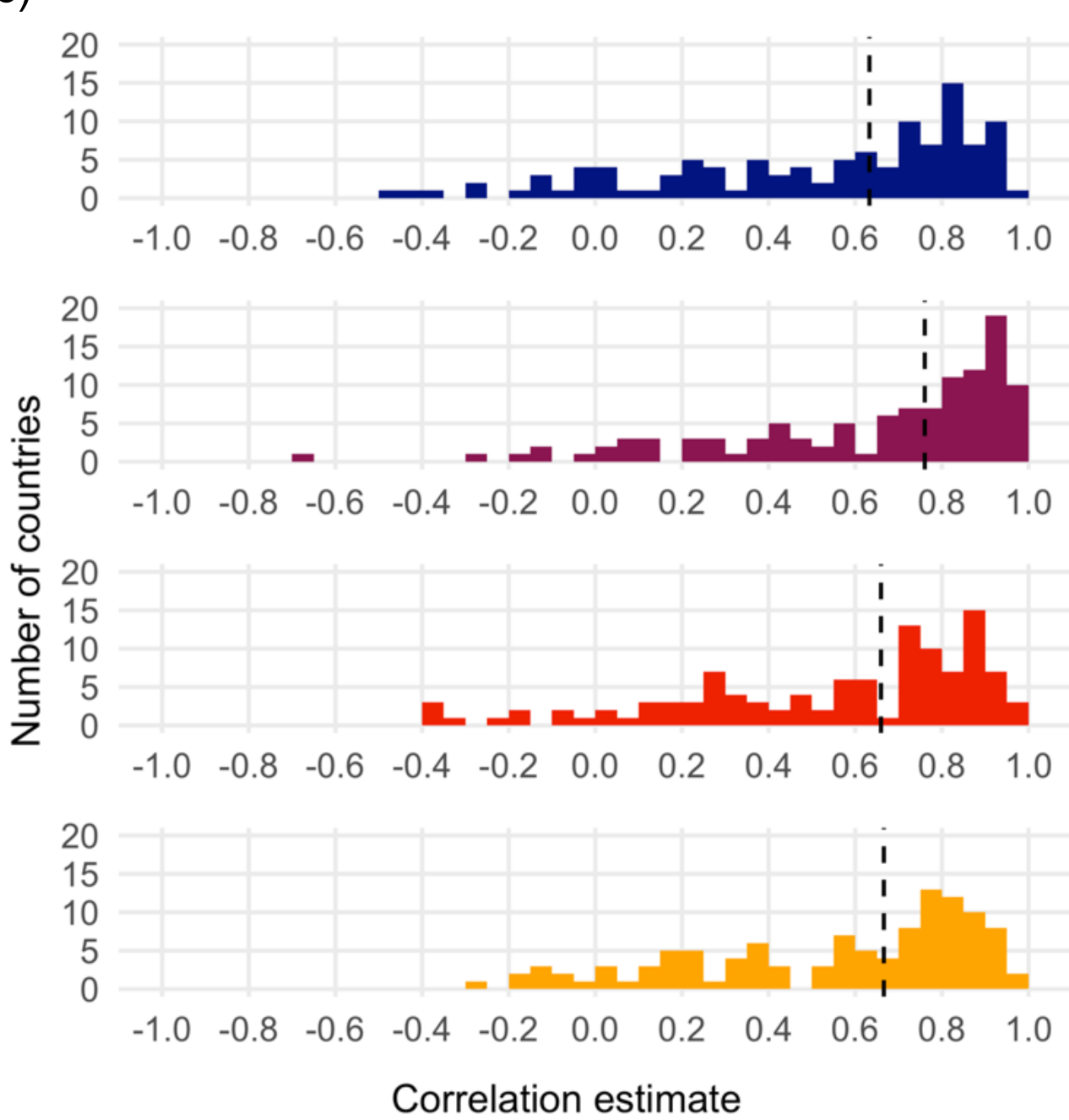

Supplementary Table 1. Survey questions, response options, and notes regarding survey changes and coding.

Covariate	Survey question/description	Response	Comments, versions and changes
Symptoms	B1: In the last 24 hours, have you had any of the following?<br><br>Fever, cough, difficulty breathing, fatigue, stuffy or runny nose, aches or muscle pain, sore throat, chest pain, nausea, loss of smell or taste, eye pain, headache.	Respondents may select none or up to 14 symptoms<br><br>1 = Yes<br>2 = No	Chills (version 3) and changes in sleep (version 6) were not included in our analyses since they were added later and this presented with many missing values.
Duration of illness	B2: For how many days have you had at least one of these symptoms?	Open response: number validation	Only asked if B1 selected symptoms count greater than or equal to 1
Community COVID-19-Like Illness (CCLI)	B3: Do you personally know anyone in your local community who is sick with a fever and at least one other symptom?	1 = Yes<br>2 = No	In version 6 this was slightly changed to "Do you personally know anyone in your local community who is sick with a fever and either a cough or difficulty breathing?"
COVID-19 testing ever received	B6: Have you ever been tested for coronavirus (COVID-19)?	1 = Yes<br>2 = No	
COVID-19 testing recently received	B7: Have you been tested for coronavirus (COVID-19) in the last ${e://Field/sympdays} days?<br>Note: sympdays is B2, but capped at 14 days.	1 = Yes<br>2 = No	Only asked if B6 is 'Yes' and B2 greater than or equal to 1 until version 4 (06/26/2020): then only asked if B6 is 'Yes'<br>This was changed (version 7) to simply "Have you been tested for coronavirus (COVID-19) in the last 14 days?"
COVID-19 positive test	B8: Did your most recent test find that you had coronavirus (COVID-19)?	1 = Yes<br>2 = No<br>3 = I don't know	Only asked if B7 is 'YES'

Social Distancing	C1_m: In the last 24 hours, have you had direct contact with anyone who is not staying with you? Direct contact means spending longer than one minute within two meters of someone or touching, including shaking hands, hugging, or kissing.	1 = Yes<br>2 = No	Surveys answering NO coded as social distancing (per survey)
Either Social Distancing or Masking	C5: In the last 7 days, how often did you wear a mask when in public?	1=All of the time<br>2=Most of the time<br>3=Some of the time<br>4=A little of the time<br>5=None of the time<br>6=I have not been in public during the last 7 days	Surveys answering 1, 2 or 6, OR those social distancing (see above) coded as either social distancing or masking (per survey)
Gender	E3: What is your gender?	1 = Male<br>2 = Female<br>3 = Prefer to self-describe: [text entry]<br>4 = Prefer not to answer	This was changed in version 6 to<br>1=Male<br>2=Female<br>3=Other<br>4=Prefer not to answer
Age	E4: What is your age?	1 = 18-24 years<br>2 = 25-34 years<br>3 = 35-44 years<br>4 = 45-54 years<br>5 = 55-64 years<br>6 = 65-74 years<br>7 = 75 years or older	
Country	Country name as used in country level aggregates	Character	Ex: United Kingdom
Date recorded	Date that the response was recorded	Date/Time	Ex: 2020-05-01, 00:04:26
Survey duration (time)	Duration of response in seconds	Integer	Ex: 72

Weight	Survey weight to adjust to FB user population	Number (float)	Ex: 1526.36144<br>115 countries are weighted.<br>Weighting documentation: https://arxiv.org/abs/2009.14675
Missing data was coded throughout the survey as follows: -99 = missing/valid skipped/invalid,<br>-77 = seen but unanswered			

Supplementary Table 2. Parameters for global model.
Specific *lgbm.LGBMClassifier* function parameters for the global model.

**Parameter**	**Specified Value**
boosting_type	gbdt
objective	binary
n_jobs	16
silent	True
max_depth	-1
max_bin	255
subsample_for_bin	200
subsample	1
subsample_freq	1
min_child_weight	1
scale_pos_weight	1
early_stopping_rounds	30
colsample_bytree	0.6
learning_rate	0.07
min_child_samples	50
min_split_gain	0.75
n_estimators	500
num_leaves	32
random_state	501
reg_alpha	1.2
reg_lambda	1.2
